# Eutrophication and *Dreissena* Invasion as Drivers of Biodiversity: A Century of Change in the Mollusc Community of Oneida Lake

**DOI:** 10.1371/journal.pone.0101388

**Published:** 2014-07-10

**Authors:** Vadim A. Karatayev, Alexander Y. Karatayev, Lyubov E. Burlakova, Lars G. Rudstam

**Affiliations:** 1 Office of Undergraduate Biology, Cornell University, Ithaca, New York, United States of America; 2 Great Lakes Center, SUNY Buffalo State, Buffalo, New York, United States of America; 3 The Research Foundation of The State University of New York, SUNY Buffalo State, Buffalo, New York, United States of America; 4 Cornell Biological Field Station, Department of Natural Resources, Cornell University, Bridgeport, New York, United States of America; Dauphin Island Sea Lab, United States of America

## Abstract

Changes in nutrient loading and invasive species are among the strongest human-driven disturbances in freshwater ecosystems, but our knowledge on how they affect the biodiversity of lakes is still limited. We conducted a detailed historical analysis of the mollusc community of Oneida Lake based on our comprehensive lakewide study in 2012 and previous surveys dating back to 1915. In the early 20th century, the lake had a high water clarity, with abundant macrophytes and benthic algae, and hosted the most diverse molluscan community in New York State, including 32 gastropod and 9 unionid species. By the 1960s, lake turbidity increased during a period of anthropogenic eutrophication, resulting in a 38% decline in species richness and a 95% reduction in abundance of native gastropods grazing on benthic algae. Following the invasion of *Dreissena* spp. in 1991 and subsequent increases in water clarity, native gastropod species richness expanded by 37% and abundance increased 20-fold by 2012. In contrast, filter-feeding unionids were unaffected by increased turbidity during the period of eutrophication but were extirpated by dreissenids. Through contrasting effects on turbidity, eutrophication and *Dreissena* spp. have likely driven the observed changes in native grazing gastropods by affecting the abundance of light-limited benthic algae. Given the high species richness and ecological importance of benthic grazers, monitoring and managing turbidity is important in preserving molluscan diversity.

## Introduction

Freshwater ecosystems occupy less than 1% of the Earth’s surface, yet contain ∼10% of all described species [1]. At the same time, 90% of the world’s population lives near fresh water, making these unique ecosystems hotspots of both biotic diversity and human activity [2]. As the global population continues to grow, biotic and abiotic processes in these ecosystems are being impacted, and in some cases profoundly altered, by anthropogenic activities [3], [4]. Our attempt to abate the precipitous decline in global biodiversity therefore places a premium on identifying the extent to which human-mediated disturbances affect the species richness of these systems.

Theory predicts that changes in environmental parameters, such as nutrient content, temperature, or turbidity, can produce regime shifts between system states with disparate biotic communities [5], [6], [7]. Disturbances can perturb systems as discrete events (“pulses”), or as continuous processes (“presses”), with the latter more likely to produce long-term ecosystem changes [8]. In lakes, perhaps the strongest and most widespread anthropogenic press disturbances have been eutrophication [9] and invasive species, particularly the spread of zebra (*Dreissena polymorpha*) and quagga (*D. rostriformis bugensis*) mussels, which act as powerful ecosystem engineers [10], [11], [12], [13].

The primary ecosystem-wide effects of nutrient loading and *Dreissena* filtering activity are changes in the magnitude, sources, and/or flow of basal energy sources. Whereas most studies have focused on the effects of these disturbances on phytoplankton and subsequent changes in offshore communities [6], [14], [15], benthic algae also constitute an important – and sometimes dominant – source of primary production [16]. In contrast to phytoplankton, benthic primary production is generally limited by light availability rather than nutrients [17], [18], [19]. Turbidity is positively affected by phytoplankton density, which increases with nutrient content in the euphotic zone and is suppressed by the presence of effective phytoplankton grazers such as *Dreissena* [5], [10], [20]. By having opposite effects on benthic and pelagic sources of primary production, nutrient loading and *Dreissena* invasion have dramatically restructured food webs across hundreds of lakes in North America and Europe [7], [20], [21]. However, we know much less about how these press disturbances will affect the species richness of freshwater ecosystems, particularly when both stressors co-occur. Furthermore, historical benthic data are rare and few studies have considered the effects of eutrophication and *Dreissena* invasion on the species richness of benthic communities.

In Oneida Lake, the largest and best studied inland lake in New York State with a strong regional economic and recreational importance, eutrophication and *Dreissena* have been the strongest, multi-decadal disturbances with profound system-wide effects. At the beginning of the 20^th^ century, F. C. Baker [22], [23] conducted one of the world’s first quantitative benthic studies and found Oneida to support the most diverse molluscan communities in the state (>42 species, including 32 gastropods). Subsequent studies replicating Baker’s sampling design were conducted in 1967 at the peak of eutrophication [24], and in 1992–95 shortly after the invasion of *Dreissena* in 1991 [25]. This produced a unique historical dataset enabling a rigorous assessment of changes in the structure and species richness of the molluscan community following eutrophication and *Dreissena* invasion.

With 350 species of mussels and clams and 703 gastropod species, molluscan diversity in North America is among the richest in the world, and constitutes a major portion of the species richness of North American freshwaters [26], [27]. At the same time, over two-thirds of all mollusc species in the region are considered at risk. Gastropods in particular are experiencing a rate of extinction 10,000 times higher than background extinction rates – the highest among any group of organisms [26]. In Oneida Lake, gastropod species richness declined by 40% from 1915 to the early 1990s following eutrophication [25], likely because of increases in turbidity. However, following reductions in nutrient loading, and particularly with the invasion of *Dreissena*, turbidity declined and benthic primary production increased [28], [29], [30]. Therefore, we hypothesized that the density, and possibly diversity, of gastropods grazing on benthic algae in Oneida Lake had increased. To corroborate this hypothesis, we conducted a lake-wide quantitative survey of the mollusc community which included historically sampled sites.

Pairing our results with previous studies spanning 96 years of data on the mollusc species and limnology of Oneida Lake, we conducted a historical analysis to (1) evaluate eutrophication and *Dreissena* invasion as drivers of molluscan diversity and community structure, (2) compare the dynamics in abundance and species richness of taxa relying on benthic (grazing gastropods) *vs.* pelagic (filter-feeding bivalves) primary production, and (3) consider the implications for the conservation of gastropod biodiversity.

## Methods

### Sample collection and processing

**Figure 1:**
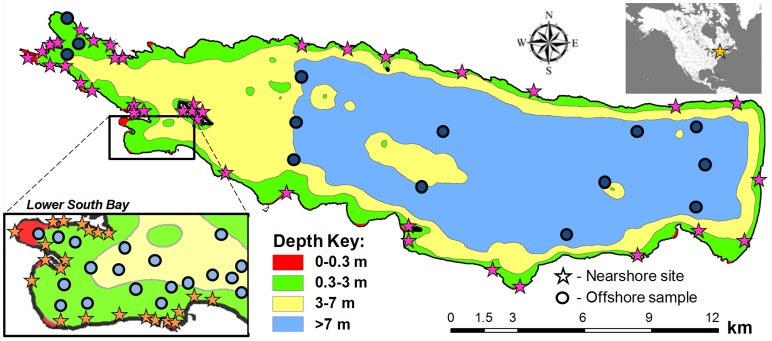
Map of 2012 sampling locations in Oneida Lake. Each nearshore site (stars) included quantitative samples at 0.1–0.3 m, 0.5 m, and 1 m depths and a qualitative sample. The inset gives site locations in Lower South Bay from which samples were collected in 1917, 1967, 1992, and in 2012, and used in the historical analysis of the mollusc community.

To assess the current diversity, density, and distribution of molluscs in Oneida Lake, we collected 221 quantitative samples, each containing 1–3 replicates, and 50 qualitative samples in June 2012 (Figure 1). We followed a unified sampling procedure, collecting samples from sites in the littoral and profundal zones across the entire waterbody. In the west end of the lake, our sites were located at all known historically sampled locations to compare the current mollusc diversity and density with those found in previous studies. These included sites from a qualitative mollusc study in westernmost parts of the lake by Baker in 1915 [22], and quantitative studies of Lower South Bay by Baker in 1917 [23] and Harman and Forney in 1967 [24]. Although studies in 1968 and the 1990s [25], [31] included qualitative samples from around the lake, we were unable to determine the locations of these sites. Therefore, our sites in the central and eastern areas of the lake were located at random intervals along the shoreline (20 sites), and along 4 north-south transects in the profundal (>7 m) zone (Figure 1). At all sites near the shoreline, we collected samples at the water boundary (0.1–0.3 m), 0.5 m, and 1 m depths to capture the diversity and distribution of molluscs in this physically heterogeneous area of the lake. In addition, molluscan species composition was examined qualitatively at each nearshore site along a 30–50 m stretch of the littoral zone down to a depth of 1 m.

Quantitative samples were collected by scraping molluscs from rocks (surface area 0.01–0.12 m^2^), while Ekman grabs (0.023 m^2^) were used to sample softer sediments. Samples of emergent macrophytes were collected by harvesting the vegetation in a ∼0.02 m^2^ area, including roots and sediments to a depth of 5 cm into the bottom. All samples were washed through a 500 µm mesh and stored in sealable plastic bags. At the lab, all dreissenids were identified to species, counted, and weighed; all other molluscs were picked and preserved in 10% neutral buffered formalin. Later, remaining molluscs were identified to species, and the density and biomass (total wet weight, with shells) in each sample was determined for every taxonomic group. Wet weight was determined by blotting off excess water and weighed using a standard (±0.01 g) or analytical (±0.0001 g, for weights <10 g) balance. Dreissenid mussels were opened with a scalpel to drain water from the mantle cavity before weighing.

All molluscs collected were identified to species level, with the exception of Sphaeriids, which were identified to family. We were only able to identify adult *Physella* to species, and were unable to determine the density of each species in samples due to the large amounts of juveniles. Therefore, we pooled *Physella* spp. species to the genus level for density and biomass estimates, but used the species level to determine the total mollusc diversity in the lake and in Lower South Bay (Table 1, S1). Although Baker [23] reported at least 26 species of sphaeriids from Oneida Lake (many of which were later determined to be synonyms), none of the later studies identified this group; therefore, we consolidate them to the family level. All current and historical data were brought to the common taxonomic resolution using the Integrated Taxonomic Index System (http://www.itis.gov).

**Table 1:** Long-term data on species richness (exotic species richness in parenthesis), density (1915–2012, m^−2^± standard error), and biomass (g m^−2^, ± standard error, lakewide 2012 only) of Oneida Lake molluscs in Lower South Bay (LSB) and lakewide (LW).

Taxa	1915–17 [22], [23]		1967–68 [24]		1992–95 [25], [72]		2012		
	LSB	LW	LSB	LW	LSB	LW	LSB	LW	LW Biomass
**Gastropoda**									
** Species richness**	26 (1)	32 (1)	10 (1)	29 (1)	13 (1)	20 (1)	23 (2)	29 (3)	
** Exotic species, m^−2^**	8.0±0.3	n.a.	435	n.a.	n.a.	n.a.	9.5±4.6	73±16	2.3±0.7 g m^−2^
** All gastropods, m** ^−**2**^	792±121	n.a.	472	n.a.	n.a.	n.a.	748±141	608±115	6.8±0.9 g m^−2^
**Bivalvia**									
**Species richness**	4 (0)	9 (0)	n.a.	9 (0)	4 (1)	6 (1)	2 (2)	2 (2)	
** Exotic species, m** ^−**2**^	0	0	0	0	n.a.	n.a.	2387±610	2420±434	458±114 g m^−2^
** All bivalves, m** ^−**2**^	288±7	n.a.	n.a.	n.a.	n.a.	n.a.	2403±610	2461±430	458±114 g m^−2^
**Total number of species**	30 (1)	41 (1)	10 (1)	38 (1)	17 (2)	26 (2)	26 (4)	31 (5)	

Note: Density data for Lower South Bay is the mean density (m^−2^) across all samples collected in each study; lakewide densities and biomass for 2012 represent averages weighted by substrate and depth areas. Sphaeriid species richness was not determined, and is excluded from the total number of species. For species-specific data, see Table S1.

### Lakewide distribution of the present community

In order to assess and compare the density and biomass of each mollusc taxon, we calculated lakewide averages (± standard error) of these parameters, weighted by the area of each substrate type (rocks, gravel, sand, silt) and depth interval (0–0.3, 0.3–3, 3–7, and >7 m) following Manly [32]. This was done by first calculating the average density and biomass of each species on every substrate within each depth interval. Each of these values were then multiplied by the proportion of the total area of the lake occupied by the given substrate type at the specific depth interval, determined using ArcGIS software (ArcGIS Desktop. Redlands, CA: Environmental Systems Research Institute ESRI 2011) and based on contour and substrate maps of the lake. The weighted lake-wide average densities and biomass were then determined by taking the sum of these values.

We tested for differences in the structure of the mollusc community across depth intervals and substrates using nonparametric multivariate statistical techniques on data matrices of species abundances in samples using PRIMER 6 (Plymouth Routines In Multivariate Ecological Research, Version 6.1.6, Primer E-Ltd. 2006). These tests of significance were based on permutation tests, combined with a general randomization approach to the generation of significance levels, allowing us to avoid the problem of spatial autocorrelation, which violates the assumption of independence of most standard statistical procedures [33]. Data were normalized via square- or fourth-root transformations, and outlying samples without any molluscs were excluded from the analysis. The similarity of the assemblage composition between all samples was summarized by calculating Bray-Curtis similarity indices (BC) ranging from 0 (no species in common) to 1 (identical samples) [34], [35]. We then used a one-way Analysis of Similarity (ANOSIM) to test the effect of substrate on the mollusc community structure across samples. ANOSIM is a resampling technique that uses permutation/randomization methods on BC similarity matrices to identify differences among groups of samples [35]. The value of the test statistic (Global *R*), ranging from 1 to −1, is a comparative measure of the degree of separation between the groups of samples being compared (values close to 1 indicate complete separation of groups; values near 0 represent little or no separation), and is thus more indicative than its statistical significance, which is limited by the number of available permutations [36]. For a complete description of the tests used see Clarke and Green [37], and Clarke [35].

### Historical analysis of Lower South Bay

The three main quantitative studies of molluscs in Lower South Bay were conducted in 1917 ([23], 165 samples), 1967 ([24], 128 samples), and during our study in 2012 (83 samples). The results of a qualitative mollusc study of the bay in 1992 are given in Table 1, but could not be used in this analysis. Although Harman and Forney [24] reported the average density for each species across all samples, only presence-absence primary data for each sample collected in 1967 were available. Therefore we were only able to compare presence-absence data of the species in individual samples in our community analysis across 1917, 1967, and 2012 using a one-way ANOSIM. Although temporal autocorrelation was a possible confounding factor in this analysis since each study sampled the same sites, this was offset by the large temporal periods between each study (∼50 years) and the conservative, nonparametric nature of ANOSIM. In a separate PERMANOVA analysis, we also found that including random effects of sites had no effect on the strength or significance of the temporal effect (authors’ unpublished data). Finally, not all of the original sites sampled in 1917 were revisited in subsequent studies, and as noted above, the analysis excluded outlier samples in which no species were found. Therefore, only ∼30% of the 222 samples in the analysis were revisited on all 3 occasions, which further reduced any spatiotemporal autocorrelation present.

To compare the density of each species across the three time periods, we had to average the densities of each taxonomic group in Lower South Bay across all samples collected in 1917 and 2012. The original study by Baker [23] spanned the entire area of the bay, and covered a range of substrates and depths. As the 1967 and 2012 studies sampled the same sites as in 1917, differences in the calculated mollusc densities across years should be representative of density changes experienced by the molluscan community in the bay. For a more rigorous analysis, we conducted a density-based comparison of the overall molluscan communities in 1917 and 2012 (where we had density data in each sample), and a similar comparison restricted to gastropods, using a one-way ANOSIM on square-root transformed data.

To test whether community dominance changed among years, we analyzed k-dominance curves and conducted “Similarity profile” permutation tests (SIMPROF routine) on the overall gastropod density data from 1917, 1967, and 2012. The k-dominance curves are determined by ranking species in decreasing order of overall abundance along the x-axis, with their relative contribution to the cumulative gastropod abundance on the y-axis. To distinguish assemblages among years we used Cluster analysis followed by SIMPROF to test for significant differences among clusters. The data was normalized for analysis by a square-root transformation [38]. All tests effects were considered significant if *P*<0.05. To visualize the differences among assemblages, we used Non-metric Multi-Dimensional Scaling (NMDS), which calculates a set of metric coordinates for each sample that most closely approximates the nonmetric distance to every other sample based on Bray-Curtis similarity indices. NMDS was found to be consistently reliable in a comparative study of ordination methods for community data [39].

As species richness is dependent on sampling effort, we estimated local species richness in Lower South Bay for 1917, 1967, and 2012 by extrapolating species accumulation curves through permutations of available samples. We also used several different nonparametric indices (Chao 1 & 2, Jacknife 1 & 2, and Bootstrap) to extrapolate the true total number of species that would be observed as the number of samples increases to infinity [40]. For 1917, 1967, and 2012 data, species richness became saturated at sample sizes between 50 and 100, a condition met by all 3 quantitative studies in Lower South Bay. All 5 indices also predicted that >85% of true species richness was detected in each study. Unfortunately, we do not know the number of samples collected in Harman’s 1992 survey [25], but considering that the author compares the results of this study with his 1967 survey, we assume that the sampling effort had to be similar and adequate. Therefore, we believe our comparison of species richness among years is valid. Using the same methods as above, nonparametric indices also predicted that >90% of true species richness was detected under 221 quantitative samples in 2012. Although we did not have sufficient data to conduct a similar analysis of lakewide species richness in other years, with the exception of the late 1960s, the number of species reported in Lower South Bay accounted for the majority of molluscan species richness in Oneida Lake (Table 1).

From historical limnological data (collection methods and data described in [29], we calculated the annual Secchi depth, chlorophyll *a*, and total phosphorus levels during the growing season by averaging weekly May-October measurements at 4 offshore stations. We then determined 5-year averages of these annual values and their standard deviation for each 5-year period. We also compared the annual mean Secchi depth readings during the growing season for years before (1970–1991) and after (1992–2011) *Dreissena* became abundant in Oneida Lake using a two-sample *t*-test assuming equal variances and a significance level α = 0.05.

### Ethics statement

We used a sampling permit (Scientific License to Collect or Possess Freshwater Invertebrates # 1390 to Lyubov E. Burlakova) issued by the New York State Department of Environmental Conservation’ Division of Fish Wildlife and Marine Resources Special Licenses Unit. Our study did not involve any protected species, and we accessed only public lands or those owned by Cornell University.

## Results

### Current state of the molluscan community

During our study, we found a total of 31 molluscan species, most of which (84%) were native to the region (Table 1, S1). Gastropods were the most diverse group, consisting of 23 native and 3 exotic species: *Bithynia tentaculata, Valvata piscinalis*, and *Cipangopaludina chinensis.* Our finding of *C. chinensis* at the entry of the New York State Canal System (Brewerton, NY) is the first record of the species in Oneida Lake. No live unionids were found, and bivalves were represented by exotic dreissenids and un-identified native sphaeriids.

The most abundant species were *D. r. bugensis* (53% of lakewide mollusc density), *D. polymorpha* (25%), and *Gyraulus parvus* (4%); molluscan biomass was strongly dominated (98%) by *Dreissena* spp. (Table S1). Due to the prevalence of *Dreissena* spp., exotic species dominated the overall molluscan density and biomass; excluding dreissenids, exotic species accounted for 12% and 33% of mollusc density and biomass, respectively. Native bivalves (sphaeriids) accounted for only 6% and 3% of lakewide mollusc density and biomass, respectively.

**Figure 2:**
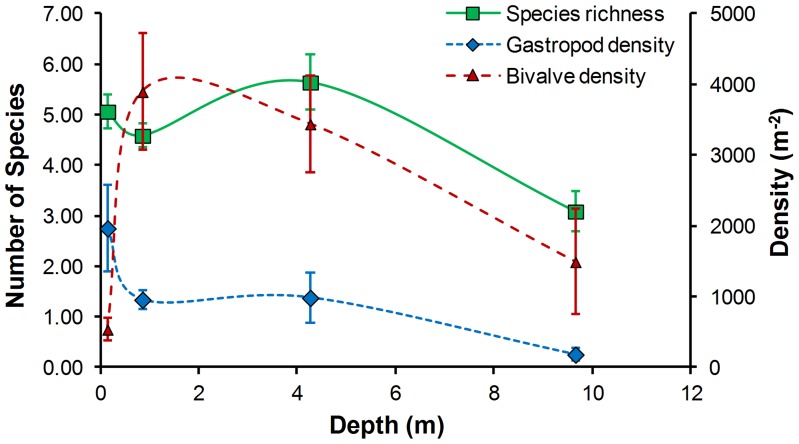
Species richness and density of molluscs at different depths in 2012 in Oneida Lake. Error bars indicate standard error.

Molluscan species richness was highest at depths between 3 and 7 m (Figure 2). Gastropod densities peaked at the water edge (0–0.3 m), whereas filter-feeding bivalves were most abundant at greater depths, and retained a strong presence in the profundal (>7 m) zone. However, as *Dreissena* abundance varied greatly within each depth interval due to differences in substrates, the effect of depth on dreissenid density was not significant. Depth intervals had a significant effect on mollusc community composition when dreissenids were excluded from the analysis (*R* = 0.12, *P* = 0.001, one-way ANOSIM). In this analysis, with the exception of the 0–0.3, 0.3–3 and 0.3–3, 3–7 depth comparisons, most pairwise tests showed strong (R = 0.33–0.67, P = 0.001) differences between depth intervals. Across substrates, molluscan diversity and density were highest on macrophytes due to large numbers of juvenile gastropods found on the plants and in the roots, whereas bivalve abundance (mostly *Dreissena*) and biomass peaked on rocky substrates (Table 2). In contrast, sandy substrates had the lowest mollusc abundance, biomass, and species richness. However, the overall effect of substrate type on community structure was low (*R* = 0.14, *P* = 0.001, one-way ANOSIM).

**Table 2:** Average (± standard error) mollusc species richness, density (m^−2^), and biomass (g m^−2^) across different substrates.

Parameter		Gravel (13)	Macro-phytes (12)	Rocks (101)	Sand (57)	Silt (38)
**Species Richness**		5.5±0.6	6.8±0.6	5.6±0.2	3.1±0.4	3.7±0.3
	**All molluscs**	4400±1967	6630±1344	6249±1232	1356±357	2158±606
**Density**	**Gastropods**	1212±437	4414±1205	1263±254	551±181	580±204
	**Bivalves**	3188±1595	2216±1084	4986±1189	805±282	1578±429
	**All molluscs**	539.7±248.6	290.8±123.3	1263.7±288.1	171.0±96.6	173.9±68.0
**Biomass**	**Gastropods**	11.7±4.4	56.7±16.3	27.6±6.4	6.7±1.6	4.9±1.1
	**Bivalves**	528.0±244.6	234.1±124.8	1236.2±288.4	164.3±96.4	169.1±68.0

Number of samples on each substrate are given in parentheses.

### Changes during period of eutrophication

At the beginning of the 20^th^ century, the Oneida Lake mollusc community included 31 native gastropods, 9 bivalve unionids, sphaeriids, and the exotic gastropod *B. tentaculata*, which at the time was a recent invader of the lake [22], [23]. Mean mollusc density in Lower South Bay was 1080±204 m^−2^, most of which was accounted by sphaeriids (26%) and 4 species of small, native gastropods (59%).

**Figure 3:**
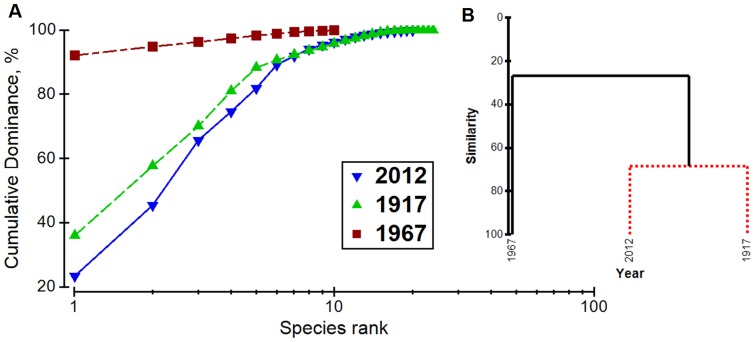
Density-based comparisons of mollusc community in Lower South Bay. A: k-dominance curve of the gastropod assemblage found in Lower South Bay in 1917, 1967, and 2012 based on species abundances across all samples in each study. B: Cluster analysis (group average) built on Bray-Curtis similarity matrix of average yearly gastropod densities. Black solid lines are significant at *P* = 0.002 (SIMPROF test).

**Figure 4:**
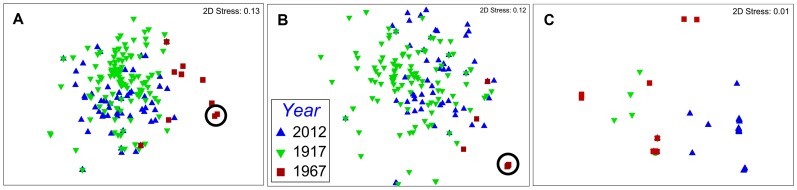
NMDS ordination plots of the molluscan community in Lower South Bay. Structure of the total molluscan community (A), gastropod (B) and bivalve (C) assemblages based on Bray-Curtis similarities of species presence-absence in samples collected in the Lower South Bay of Oneida Lake collected in 1917 [22], [23], 1967 [24], and 2012. The circled points in (A) and (B) represent 33 samples from 1967 in which only *B. tentaculata* was found.

By 1967, composition of the gastropod assemblage was drastically altered (Figures 3, 4b). Overall, the average density of gastropods in Lower South Bay declined by 41%, including a twenty-fold (95%) decline in density of native gastropods [24] (Table 1). In contrast, the density of the exotic *B. tentaculata* increased from 8.3 to 435 m^−2^, and comprised 92% of all molluscs in Lower South Bay in 1967. No change in the frequency of occurrence or lakewide diversity of unionid bivalves was detected by Harman and Forney [24], but the frequency of occurrence of *Pisidium* spp. in samples collected at the same sites dropped from 62% in 1917 to 10% in 1967.

Lakewide molluscan species richness declined by only 3 species from 1917 to 1967, but in Lower South Bay the number of gastropod species dropped by 62%, with 16 species locally extirpated (based on 115 samples; Table 1) [24]. A much stronger decline in lakewide gastropod species richness (38% since 1968) was detected in lakewide surveys during 1992–1995 [25], [31]. Species lost included the regionally rare *Acella haldemani*, *Lymnaea stagnalis*, and *Somatogyrus depresus*, which have not returned as of 2012.

### Changes following *Dreissena* invasion

With the invasion of *D. polymorpha* in 1991, species richness of gastropods on a lakewide scale increased by 45% in 2012 compared to 1995 (Table 1). Species newly present in 2012 included 5 of the 14 gastropod species lost during the period of eutrophication and not reported since 1915 (Table S1). In addition, 6 species present in 2012 (including 3 exotic and 3 native to the region) were not reported during previous lakewide studies. Native gastropod species richness in Lower South Bay had increased by 83% between 1993 and 2012, and the mean gastropod density became similar to that reported by Baker [23] (792±121 m^−2^ in 1917 *vs.* 749±141 m^−2^ in 2012, mean ± standard error). In contrast, *B. tentaculata* abundance declined by 98% from 1967 to 2012, and its densities are now similar to those in 1917.

In contrast to gastropods, unionid species richness dramatically declined following the *D. polymorpha* invasion. Three species were lost from the lake by 1995, and lakewide surveys found no live unionids in the lake both in 1997 [25] and in 2012.

### Community analyses of Lower South Bay across years (1917–2012)

Mollusc communities in Lower South Bay in 1917 and 2012 were not significantly different (*P* = 1.0; 68% similarity; SIMPROF test based on densities in Table S1, Figure 3b), while both the 1917 and 2012 years differed significantly from 1967 (*P* = 0.002; 27% similarity). Moreover, the dominant groups in 1917 and 2012 were similar: 7 of the 10 most common species in 1917 were also among the 10 most common species in 2012.

Presence-absence based analysis comparing the 1917, 1967, and 2012 datasets showed strong differences in the gastropod assemblages among years (*R = *0.462, *P* = 0.001, one-way ANOSIM). The largest differences were found among the assemblages in 1967 and 1917 (*R* = 0.697, *P* = 0.001, pairwise comparisons after one-way ANOSIM; Figure 4b), and between 1967 and 2012 (*R* = 0.742, *P* = 0.001). In contrast, no large differences were found between gastropod assemblages in 1917 and 2012 (*R* = 0.148, *P* = 0.001). Due to the relatively high abundance and species richness of gastropods, very similar trends are seen in the ANOSIM comparing the overall molluscan community across years (Figure 4a).

Similarly to presence-based comparison, density-based ANOSIM of the gastropod assemblage revealed small differences (*R* = 0.131, *P* = 0.001, one-way ANOSIM) between 1917 and 2012. Larger differences were found between densities and structure of overall molluscan communities (i.e., including bivalves) between these years (*R* = 0.382, *P* = 0.001, one-way ANOSIM), driven mainly by the relatively strong presence of *Dreissena*, the loss of unionid species, and a 94% decline in sphaeriid density.

## Discussion

Over the past century, the molluscan community of Oneida Lake has undergone two dramatic transformations, the latter of which resulted in a community relatively similar to its original state. These community shifts were concomitant with major shifts in ecosystem turbidity caused by cultural eutrophication and *Dreissena* introduction, and were primarily characterized by changes in the diversity and density of grazing gastropods. In contrast, filter-feeding unionids were unaffected by increased turbidity associated with eutrophication and extirpated by *Dreissena* spp. These dynamics highlight turbidity as a likely master variable in regulating the abundance and diversity of benthic grazers via bottom-up effects.

### Coupling of mollusc abundance and diversity with turbidity

**Figure 5:**
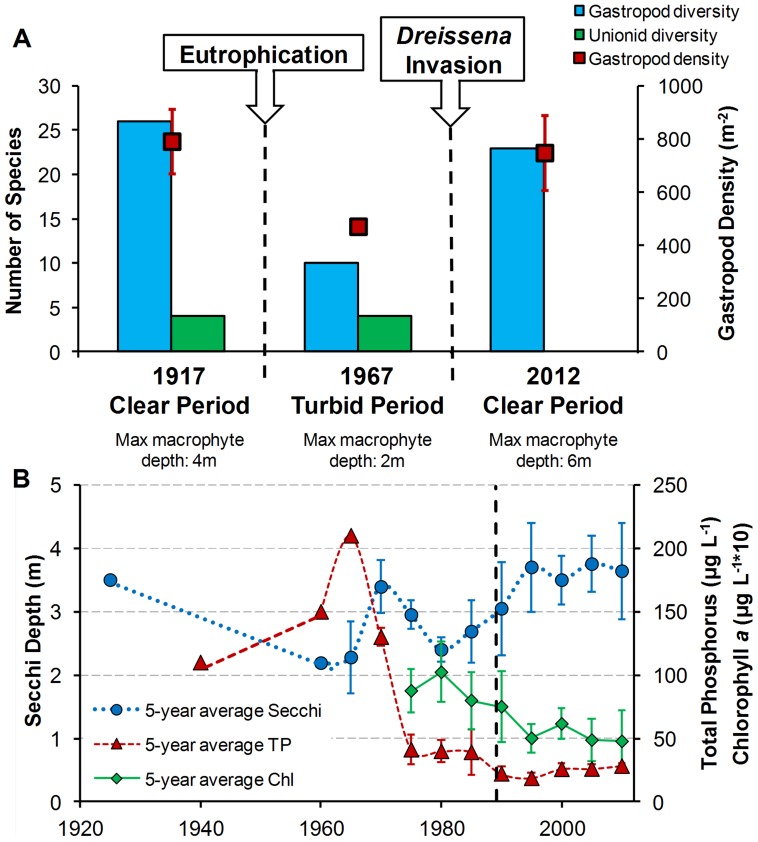
Long term changes in the mollusc community and lakewide limnological parameters. A: Gastropod and unionid species richness (bars) and gastropod average density (± standard error, where available) in Lower South Bay in 1917 [23], 1967 [24], and 2012 (our study). B: Growing season (May-October) values of total phosphorus, chlorophyll *a*, and Secchi depth in Oneida Lake from 1927 to 2011, averaged across 5-year periods. Where available, error bars indicate standard deviation of annual growing season averages. Vertical dashed line indicates timing of *D. polymorpha* invasion. Data from Muenscher [73], Greeson [74], and Rudstam [29].

Although considered a productive lake already in the 1800s, Oneida Lake water clarity was high in the early 1900s, with abundant emergent aquatic vegetation, benthic algae, and submerged macrophytes [41]. Starting in the 1920s, land use changes increased nutrient loading and sediment runoff. Total phosphorus peaked in the late 1960s, concurrent with a strong increase in turbidity, and in Lower South Bay the coverage of aquatic vegetation declined from the 4 m contour in 1955 to the 2 m contour in the 1960s [42] (Figure 5).

The highly diverse gastropod assemblage present in 1915–1917 saw a strong decline during the turbid period (Figure 5b). As the majority of the native gastropods were benthic grazers [43], we attribute this change primarily to a decrease in submerged macrophytes and especially in periphyton and benthic algae – the primary food source for most native gastropods in the lake. A decline in benthic algal biomass with increasing turbidity due to eutrophication has been shown for numerous lakes [19]. Previous studies have observed reduced diversity and/or density of benthic macroinvertebrates in more turbid lakes [44], [45], [46]. At the same time, densities of filter-feeding unionids were unaffected, and the exotic gastropod *B. tentaculata –* which is both a grazer and a filter-feeder [47] – dramatically increased in abundance. Following the improvement of sewage treatment facilities in the lake’s watershed, nutrient input began to subside in the late 1970s. However, Secchi depth increased only slightly (∼10%) from the 1960s to the late 1980s (Figure 5b), and by the early 1990s gastropod species richness had declined by 38% from 1915–1917.

From 1992, water clarity in Oneida Lake increased as *D. polymorpha* became abundant, with significant increases in average May-October Secchi depth compared to pre-invasion years (*P*<0.001, *t*-test; Figure 5b). These changes have been attributed to *Dreissena* filtering activity rather than to reductions in nutrient loading [28], [48], and similar increases in water clarity following dreissenid invasions have been widely reported [7], [20], [49]. As light levels reaching the benthic community increased, the maximum depth at which macrophytes were reported nearly doubled by 2002 [28], likely accompanied by an expansion of the area covered by benthic algae. By 2004, the primary production of benthic algae had increased significantly, and its inter-annual variability decreased [30]. Consistent with a bottom-up regulation, Mayer et al. [48] detected an increase in shallow-water benthic grazers (gastropods and amphipods) within 5 years of the *Dreissena* invasion, and long-term studies of fish species in the upper trophic levels found a shift in energetic reliance from phytoplankton to benthic primary production [50]. These trends have continued with the invasion of *D. r. bugensis* around 2005 [51], which often reaches its peak densities a decade after introduction to waterbodies first invaded by *D. polymorpha* [52].

We argue that the recovery of gastropod abundance and species richness was primarily driven by ecosystem-wide reductions in turbidity which expanded the main energy source of benthic grazers. In addition, several previously described, local-scale dreissenid impacts on benthic grazers could have contributed to this recovery. These include nutrient subsidies to benthic biofilms and deposit feeders through mussel excretions and through increases in deposition of organic and inorganic material [10], [49], [53], [54], [55]. *Dreissena* shells also increase the area of hard substrates available for algal growth and increase habitat complexity, thereby reducing the foraging success of predators [10], [49], [53], [55], [56].

As gastropods dominate the diversity and abundance of native molluscs, our historical analysis provides strong evidence to suggest that dreissenids have facilitated a shift in mollusc community structure towards that observed before eutrophication in 1915–1917 (Figure 4). This contrasts with the concept of invasional meltdown, which posits that the ecological effects of invasive species favor the establishment of other invasives [57], [58]. Densities of the exotic *B. tentaculata*, which dominated the gastropod assemblage at the peak of eutrophication, have dramatically declined with the invasion of *Dreissena*, while the 2 newly introduced exotic gastropods have thus far remained at low densities. This suggests that, while strong perturbations of an ecosystem from its natural state can promote rare or exotic species, disturbances in the form of species invasions *per se* do not necessarily favor non-native taxa [59].

The impacts of eutrophication and dreissenids on filter-feeding unionids differed strongly from the effects on grazing gastropods (Figure 5). During the turbid period, no changes in lakewide unionid diversity or frequency of occurrence in Lower South Bay were observed; however, the invasion of *D. polymorpha* resulted in a strong decline of unionids, which have not been reported in the lake since 1995 [25]. Similar declines in unionid diversity following dreissenid invasion have been documented across numerous lakes in North America and Europe [60], [61], [62].

### Implications for conservation

Most gastropod species extirpated from Oneida Lake by the 1990s first experienced strong declines in abundance by 1967, although only 3 species were lost at that time (Table 1). Continuing eutrophication and high turbidity likely led to the loss of more species by the 1990s, suggesting that the duration of an environmental disturbance can be an important factor affecting community diversity and structure [63], [64]. While eutrophication often manifests as a multi-decadal press disturbance, these trends indicate that timely and sufficiently strong regulations of nutrient loading can reduce the negative consequences for the species richness of gastropods, which are often the principal grazers in aquatic habitats [26]. As grazers link benthic primary production – a major (and sometimes dominant) energy source – with the upper trophic levels [16], efforts to preserve gastropods are closely linked with the conservation of biodiversity and ecosystem structure of lakes. This is of particular importance in shallower, more numerous lakes in which changes in turbidity occur more rapidly [19], biodiversity is higher [65], [66], [67], and where the relative importance of benthic primary production as an energy source is greatest [16], [19].

In ecosystems where the effects of an environmental disturbance are reversed, an important consideration in the recovery of species richness is the presence of potential donor populations and their connectivity with the perturbed system [68]. Genetic studies of gastropods have shown or suggested such metapopulation dynamics between waterbodies as the factors driving genetic diversity and species persistence [69], [70], [71]. In Oneida Lake, four of the five gastropod species lost during the period of eutrophication and which recovered by 2012 are relatively common in central New York [47] (Table S1). In contrast, all 3 regionally rare (and now listed as imperiled) gastropod species originally present in 1917 have failed to recover. A low number of donor populations hampering the recolonization of disturbed waterbodies may have contributed to the loss of rare species, which in North America account for 92.5% of the freshwater gastropods already extinct [26]. This highlights the importance of conserving species presence on a regional scale rather than on a local scale of individual waterbodies.

## Supporting Information

Table S1 **Mollusc species reported from Oneida Lake.**
(PDF)
