# Supplementary material for: Eutrophication and Dreissena Invasion as Drivers of Biodiversity: A Century of Change in the Mollusc Community of Oneida Lake
Source: PLoS One. 2014 Jul 10;9(7):e101388. doi: 10.1371/journal.pone.0101388 (PMC4092058; doi:10.1371/journal.pone.0101388)
Supplement: Table S1 — Mollusc species reported from Oneida Lake. (PDF) [file pone.0101388.s001.pdf]

Table S1. Mollusc species reported from Oneida Lake. Presence-absence (+/-), density (m^{-2} ±standard error, where available), and biomass (mg m^{-2} ±standard error, lakewide 2012 only) data given for Lower South Bay (LSB) and lakewide.

	**1915-17** [22, 23]		**1967-68** [24]		**1992-95** [25, 72]		**2012**		
	LSB (1917)	Lake-wide	LSB (1967)	Lake-wide	LSB (1992)	Lake-wide	LSB	Lakewide Density	Lakewide Biomass
**Gastropods**:									
Acella haldemani [3, 4]	0.3±0.3	+	-	+	-	-	-	-	-
Amnicola limosus	171.5±26.0	+	-	+	-	+	150.3±34.4	101.8±27.1	781±172
Amnicola pupoideus	-	-	-	-	-	-	1.6±1.2	1.6±1.0	11±7
Bithinya tentaculata [1]	8.3±4.3	+	435.0	+	+	+	8.4±3.7	77.2±15.5	2251±640
Campeloma decisum [3]	4.5±2.0	+	-	+	-	-	-	-	-
Cipangopaludina chinensis [1]	-	-	-	-	-	-	-	+	+
Elimia livescens	5.7±2.2	+	2.0	+	+	+	2.1±1.4	2.8±1.7	132±59
Elimia virginica [2]	-	-	1.0	+	-	-	3.8±1.8	1.0±0.3	310±125
Ferrissia parallela	6.0±2.2	+	-	+	+	+	5.2±2.9	1.7±0.5	6±2
Ferrissia rivularis [3]	-	+	-	+	-	-	-	-	-
Fossaria obrussa	0.3±0.3	+	-	+	+	+	1.6±1.2	3.2±1.2	22±8
Gyraulus crista	-	-	-	-	-	-	0.5±0.5	0.2±0.2	2±2
Gyraulus circumstriatus	-	-	-	-	-	-	7.6±4.9	0.8±0.5	4±3
Gyraulus deflectus	8.1±2.5	+	1.0	+	+	+	53.6±25.3	16.6±3.0	55±10
Gyraulus parvus	97.7±18.1	+	5.0	+	+	+	175.2±60.8	136.5±60.6	228±38
Helisoma anceps	10.8±4.0	+	1.0	+	+	+	3.1±1.6	3.0±1.9	10±7
Laevapex fuscus	0.6±0.5	+	-	+	+	+	7.1±3	1.7±0.6	11±4
Lymnaea stagnalis [3, 4]	-	+	-	-	-	-	-	-	-
Physella spp.	58.0±15.8	n.a.	13.0	n.a.	n.a.	n.a.	111.8±37.2	100.1±41.8	518±94
Physella ancillaria	+	+	+	+	-	-	+	+	+
Physella gyrina	-	+	-	+	-	-	-	-	-
Physella heterostropha	-	-	-	+	-	-	+	+	+
Physella integra	+	+	-	+	+	+	+	+	+
Planorbella campanulata	7.2±1.9	+	-	+	-	+	-	-	-
Planorbella trivolvis	5.1±2.7	+	-	+	+	+	20.6±8.8	12.8±2.2	394±56

Pleurocera acuta [2]	-	+	-	-	-	-	-	0.6±0.2	331±135
Pomatiopsis cincinnatiensis	2.4±1.5	+	7.0	+	-	+	-	0.02±0.02	1±1
Probythinella emarginata [3]	0.2±0.1	+	-	-	-	-	-	-	-
Promenetus exacuous [2]	12.9±3.1	+	-	-	-	+	1.2±0.8	1.3±0.8	8±4
Pseudosuccinea columella [3]	1.0±0.9	+	-	+	-	+	-	-	-
Pyrgulopsis lustrica	285.6±80.4	+	3.0	+	+	+	18.2±7.5	2.8±0.8	29±11
Somatogyrus depresus [3, 4]	0.02±0.02	+	-	-	-	-	-	-	-
Stagnicola catascopium [2]	86.8±18.2	+	-	+	-	-	67.4±26.3	27.3±7.5	475±110
Stagnicola elodes	-	+	-	+	+	+	-	0.1±0.1	8±5
Stagnicola emarginata [3]	-	+	-	+	-	-	-	-	-
Valvata lewisi [2]	-	+	-	+	-	-	0.5±0.5	1.6±1.1	45±38
Valvata piscinalis [1]	-	-	-	-	-	-	1.0±1.0	2.0±1.8	71±63
Valvata sincera	0.6±0.3	+	-	+	-	+	-	6.0±3.9	14±8
Valvata tricarinata	18.2±5.5	+	4.0	+	-	+	53.9±25.3	109.2±39.0	1152±441
Viviparus georgianus	0.01±0.01	+	-	+	+	+	+	+	+
Total gastropod species:	26 (1)	32 (1)	10 (1)	29 (1)	13 (1)	20 (1)	23 (2)	29 (3)	
**Bivalves:**									
Dreissena polymorpha [1]	-	-	-	-	+	+	923.8±194.2	780.5±158.9	63278±12304
Dreissena r. bugensis [1]	-	-	-	-	-	-	1462.8±500.7	1635.0±395.4	397662±112597
Actinonaias ligamentina	-	+	n.a.	-	-	-	-	-	-
Alasmidonta undulate	-	+	n.a.	+	-	-	-	-	-
Elliptio complanata	15.0±3.6	+	+	+	+	+	-	-	-
Lampsilis radiata	3.0±0.6	+	n.a.	+	+	+	-	-	-
Leptodea fragilis	-	-	n.a.	+	-	+	-	-	-
Ligumia recta	-	-	n.a.	+	-	-	-	-	-
Margaritifera margaritifera	-	+	n.a.	+	-	-	-	-	-
Pisidium spp.	284.7±97.7	+	n.a.	n.a.	n.a.	n.a.	16.0±4.5	41.3±13.3	202±58
Potamilus alatus	-	-	n.a.	+	-	+	-	-	-
Pyganodon cataracta	0.3±0.1	+	n.a.	-	-	-	-	-	-
Pyganodon grandis	0.4±0.2	+	+	+	+	+	-	-	-
Strophitus undulatus	-	+	n.a.	+	-	-	-	-	-

Villosa iris	-	+	n.a.	-	-	-	-	-	-
Total bivalve species:	4 (0)	9 (0)	n.a.	9 (0)	4 (1)	6 (1)	2 (2)	2 (2)	
**Total species:**	**30 (1)**	**41 (1)**	**10 (1)**	**38 (1)**	**17 (2)**	**26 (2)**	**26 (4)**	**31 (5)**	

Note: Density data for Lower South Bay is the mean density (m^{-2}) of each species across all samples collected in each study; lakewide densities and biomass for 2012 represent averages weighted by substrate and depth. For quantitative studies, + indicates a species found in qualitative samples. n.a. indicates a species was not sampled (or not recorded) in study. Total species tallies exclude sphaeriids, and denote the number of exotic species in parentheses.

[1] - species exotic to the region; [2] - gastropod species extirpated during eutrophication which has recovered by 2012; [3] - gastropod species which has not recovered following eutrophication; [4] - species classified as threatened/endangered by Johnson et al. [26]
